# Oncology practice in the COVID-19 pandemic: a report of a Nigerian expert panel discussion (oncology care in Nigeria during the COVID-19 pandemic)

**DOI:** 10.11604/pamj.2020.36.153.23662

**Published:** 2020-07-06

**Authors:** Adeniyi Adedayo Olabumuyi, Musa Ali-Gombe, Olusegun Abayomi Biyi-Olutunde, Olumide Gbolahan, Chinenye Oluchi Iwuji, Adedayo Olufemi Joseph, Nwamaka Ngozika Lasebikan, Babatunde Olutoye Ogunnorin, Adebowale Emmanuel Omikunle, Omolola Salako, Abdulazeez Salawu

**Affiliations:** 1 Radiation Oncology Department, University College Hospital, Ibadan, Nigeria,; 2 Department of Radiology, College of Medical Sciences, Gombe State University, Nigeria,; 3 University of Port-Harcourt Teaching Hospital, Port-Harcourt, Rivers State, Nigeria,; 4 University of Alabama, Birmingham, USA,; 5 Oncology Department, Leicester Royal Infirmary, Leicester, UK,; 6 NSIA-LUTH Cancer Centre, Lagos University Teaching Hospital, Lagos, Nigeria,; 7 Department of Radiation Medicine, University of Nigeria Teaching Hospital, Enugu, Nigeria,; 8 Radiation Oncology Department, University of Ibadan, Ibadan, Nigeria,; 9 Breast Cancer Squad, ROCHE Products Nigeria Ltd. Nigeria,; 10 College of Medicine, University of Lagos, Lagos, Nigeria,; 11 Nottingham University Hospitals, NHS Trust, UK

**Keywords:** COVID-19, cancer, Low- and middle-income countries, Personal protective equipment, guidelines

## Abstract

Since the first case of COVID-19 and its progression to a pandemic, healthcare systems the world over have experienced severe difficulties coping with patient care for both COVID-19 and other diseases most especially non communicable diseases like cancer. These difficulties in Low- and middle-income countries (LMICs), especially in Sub-Saharan Africa including Nigeria, are myriad. These LMICs are already bedeviled weak health systems, ill equipped cancer treatment centers, with outdated machines and grossly inadequate numbers of oncologists required to treat patients with cancer. As a result of these challenges coupled with unclear guidelines on how to manage cancer patients in the wake of the COVID-19 pandemic, 11 key Nigerian opinion leaders had a consensus meeting to identify challenges and possible workable solutions on continuing cancer care during the COVID-19 pandemic. The discussion highlighted ethical issues, barriers to continuing cancer care (such as lockdown, fear of contracting disease, downscaled health services) and resource constraints such unavailable personal protective equipment. Yet, practical solutions were proffered such as necessary protective measures, case by case prioritization or de-prioritization, telemedicine and other achievable means in the Nigerian setting.

## Introduction

Since the first case of COVID-19 and its progression to a pandemic, healthcare systems have experienced severe difficulties coping with patient care. As a result, there are growing concerns regarding the capacity for low- and medium-income countries (LMICs) to cope with a surge of severe COVID-19 cases [1]. Furthermore, the economic losses experienced has taken its toll on the ability to procure healthcare equipment and materials needed to cope with the rising incidence, admissions, critical care and mortalities associated with COVID-19 [2]. Medical centres have had to defer elective procedures such as dental, ear, nose and throat (ENT) and plastic surgery procedures to preserve resources such as hospital beds and ICU space to manage COVID-19 patients. In other centres, entire emergency rooms have been filled to capacity with COVID-19 patients, requiring makeshift overflow areas in parking lots, or factory and sports arena for the treatment of COVID-19 patients [3-6]. In spite of this, there are other health challenges besides COVID-19. Some of these health challenges require treatment even in the wake of the COVID-19 pandemic. One such disease that requires on going treatment, follow-up and procedures is cancer. The COVID-19 pandemic does pose a real threat to cancer care world over.

In some countries, patients with cancer are not able to receive care from their care givers because such facilities are filled to capacity with COVID-19 patients. In the unfortunate event that a COVID-19 patient with cancer requires ventilator support from severe respiratory distress, anecdotal evidence has shown that they are less favoured to receive such essential care due to the cancer co-morbidity [7]. Other challenges besides those above include: which patient should be prioritized or deprioritized for treatment? Which procedure (surgery, radiotherapy and chemotherapy) should continue or be deferred? And what precautionary steps should be taken to protect the health worker and patient during treatment? Unfortunately, there are no straightforward answers to these questions. The answers differ from one country to the other and from one health centre to the other. In LMICs, including Nigeria, the challenges go beyond what has been described. These LMICs are already bedeviled with weak health systems, ill equipped cancer treatment centers, with outdated machines and grossly inadequate numbers of oncologists required to treat patients with cancer. Due to this inability to arrive at a consensus on how to manage cancer patients in the wake of the COVID-19 pandemic, 11 key opinion leaders had a consensus meeting to identify challenges and practical solutions on continuing cancer care during the COVID-19 pandemic. The discussion was to highlight the challenges and barriers to cancer care in the Nigerian setting during the COVID-19 pandemic and proffer possible solutions that could be adopted by cancer treatment centres across the country.

## Methods

The eleven discussants were invited to a Zoom meeting which took place on the 30^th^of March at 5: 30pm and lasted for 1 hour 19 minutes. All the discussants were Nigerian oncologists; 8 were practicing in Nigeria, 2 in the United Kingdom; and 1 in the United States. Among the eight that practice in Nigeria, 5 practice in south-western (SW), 1 in the northern (N), 1 in the south-eastern (SE), and 1 in the south-southern (SS) part of Nigeria. They were all purposively selected to capture prevailing practice within the geographic zones that divide the country. The oncologists from Nigeria were ascribed numbers 1-8, those in the UK, 9-10 and the one in US was 11. The discussion focused on policies regarding the care of oncology patients in the COVID-19 era, in the Nigerian setting rather than individual case management. Case vignette approach was used to ensure the policies placed individual patient care in perspective. Two hypothetical case scenarios preceded the discussion followed by a poll on the options the discussants would take.

**Scenario 1:** a 66-year-old man with metastatic hormone refractory prostate cancer, whose disease had progressed on Zoladex. He is currently on intravenous Docetaxel, and has had two cycles. He lives in a different region of the country from his oncologist and has to commute by road for over 4 hours. He calls his oncologist two days prior to his due date for his third cycle of the treatment asking if he can travel over to receive the third cycle. What is the next best step to take? A) Tell him there will be no chemotherapy administration because of the COVID-19 pandemic and he should call you after the outbreak is controlled. B) Refer him to an oncologist in his locality. C) Ask him to come over for the chemotherapy as it is business as usual. D) Convert his treatment to Abiraterone (an oral drug).

**Scenario 2:** a 46-year-old widow and single mother of two diagnosed with triple negative breast cancer T3N1M0 who is yet to commence any treatment. She lives in the same locality as her oncologist who saw her the previous week and obliged her to commence neoadjuvant chemotherapy. She calls at 6am to inform her oncologist she is coming over for her chemotherapy as planned but wants to know the hospital policy regarding accompanying relatives as she wants to come with her two teenage daughters. What is the next best step to take? A) Tell her the hospital has no objections to her daughters accompanying her. B) Tell her there will be no chemotherapy administration because of the COVID-19 pandemic and she should call you after the outbreak is controlled. C) Ask her to come over for the chemotherapy, but her daughters cannot accompany her. D) Tell her that her treatment plan has changed to her having surgery first before the chemotherapy.

## Results

**Scenario 1:** the majority, 67%, chose option “B” to “refer him to an oncologist in his locality.”

**Scenario 2:** the majority, 50%, chose option “C” to “ask her to come over for the chemotherapy, but her daughters cannot accompany her.”

The polls show majority of the discussants would want cancer care to continue in spite of the challenges the COVID-19 pandemic poses. A quote during the discussion that encapsulates this tendency is: *“when this whole COVID thing is over make sure that more people have not died from the fear of COVID than from COVID itself I think that oncology is one of the places that we have to strike this balance very carefully.” (Oncologist 3, UK)*

### Barriers

In spite of this, the discussants identified existential barriers to cancer care in Nigeria due to the COVID-19 pandemic.

**Lockdown/Curfew:** some states like Lagos and Ogun states have enforced a total lockdown, which started on the 30^th^of March 2020, to curb the spread of the virus. While other states such as Oyo state enforced a dusk to dawn curfew from 7pm to 6am. One of the discussants said *“There is a curfew that will start tonight and more than 50% of the patients already stopped coming”* (Oncologist 3, SW Nigeria). Another discussant noted, regarding one of the hypothetical case scenarios, that *“if this patient is coming* (from out of state), *she won´t be able to, even if she comes in, she won´t be able to stay long.”* (Oncologist 6, SE Nigeria).

**Patients fear contracting COVID-19 from health workers:** apart from the lockdown/curfew which limits patients from accessing cancer care at oncology centres. Some patients have refused to come for fear of contracting the virus. For instance one of the discussants practicing in Lagos said that *“they are scared of the virus and they believe the hospital is a point of transmission for them Naturally our patients have dropped and they are not going to come.”* (Oncologist 3, SW Nigeria).

**Outpatient and Surgery:** due to epidemiological spread of COVID-19 within certain hospitals, the management of some hospitals scaled down services such as outpatient services and elective surgeries. Cancer outpatient clinics and chemotherapy clinics are categorized as outpatient clinics. Hence, patient evaluation, follow up, chemotherapy administration and radiotherapy procedures could not proceed as usual. One of the discussants said *“there is possibility that there might be a delay in treatment, all the out-patient departments, elective surgery, chemotherapy, (and) radiotherapy have been shut down.”* (Oncologist 1, SW Nigeria). On the other hand another oncologist said *“our out-patient facilities are not locked down but we are selecting, patients that are critically ill* (In addition,) *chemotherapy is given to patients* (if) *it will actually make a difference in their patient-journey. Elective* (surgery) *cases have been cancelled but there is also room for* (cases that are) *potentially curative and we could see as an emergency. ”* (Oncologist 6, SE Nigeria).

Similarly, another oncologist noted that *“what we decided to do is suspend brachytherapy because patients come from all over the country so we have decided to suspend taking new patients till things improve.”* (Oncologist 8, N Nigeria). Delaying chemotherapy for up front surgery is an acceptable solution. Yet the barrier to this option is surgeons have stopped elective procedures. A discussant said *“even patients who have been initially booked for surgery have been asked to go away and I do hear you say that (upfront) surgery is okay but if your surgeons are unwilling to operate what then do you do?* (Oncologist 7, SS Nigeria). Another discussant said *“we will usually have an MDT for this* (patient) *and I suspect we will offer her surgery first. The surgeons will look at her that she is young and this is a potentially curable intervention and we will go ahead.”* (Oncologist 6, SE Nigeria).

**Toll free numbers:** the government has made toll-free numbers available for anyone who has been exposed to call so as to be attended to appropriately. However, there were doubts as to its efficiency by some discussants. An oncologist stated that *“I had my own fair share of this COVID symptoms, when I did the COVID checker checklist I was high risk. I called all the numbers and I couldn´t get through.”* (Oncologist 3, SW Nigeria). Another oncologist also said *“I have tried those numbers myself and I didn´t get through to them when I thought;* (the way I am) *feeling could I possibly be* (infected).” (Oncologist 7, SS Nigeria).

**Precautions, protection of health worker and patients:** the HIV/AIDS pandemic birthed the ‘universal precautions´. With community spread of COVID-19 pandemic, should one treat every patient as a possible COVID-19 patient? One of the opinions was *“we absolutely have to consider all patients COVID patients but we also have to be practicable about what we can do. In our hospital, what we do is that we have the mask gowns and gloves on as a minimal for any patients you have to see.”* (Oncologist 10, UK). However, another oncologist painted a different picture where he/she practices. Saying *“that we had to have a shouting match at work today regarding availability of basics like face masks and other protective gears that we will like to have. We have such gross limitation with even bare basics. My basic concern is to even have enough for health workers not to mention patients.”* (Oncologist 7, SS Nigeria). Another oncologist interjected *“I think that is a clear case, if you do not have what you would require to keep you safe you should not be there.”* (Oncologist 3, SW Nigeria) An oncologist continued *“that was what I was saying at work, if we have no personal protective equipment (PPE), we have no business being at work but then hospital management is insisting everyone should be at work.”* (Oncologist 7, SS Nigeria).

However, someone else indicated *“most us will get to the point where we will run out of masks... in every part of the world. The ideal masks are N95 masks*, (and) *many parts of the world don´t even have enough of those for their emergency rooms. At some point we have to deal with the reality that we will not have the appropriate protective gears and we still have to* (work).” (Oncologist 11, USA). The question was posed, what would be considered the appropriate minimum protective gear? The discussants concluded that *“a face mask* (not necessarily N95), *conform gloves during the encounter for procedures, and if available; surgical gowns will suffice”*. (Oncologist 3, SW Nigeria). The discussants also added *“it is not enough to have all these for the physicians but the patient must also be masked as well.”* (Oncologist 6, SE Nigeria). Furthermore a discussant reiterated *“that there really is a possibility, again, that we will run out of masks. So far we try to get everybody to wear a facemask; at least one party should wear a facemask, either the practitioner or the patient. If both of you can have masks then that is fine.”* (Oncologist 11, USA).

**Ethics:** this discussion raised ethical issues. In responding to the question regarding precautions and minimum acceptable and practicable protection against COVID-19, one of the oncologists asked *“If it was us that needed cancer treatment, would we be happy for nothing to be available at all until a pandemic that might take months if not longer to resolve gets resolved?”* (Oncologist 9, UK). The discussant further stated that *“I think us having a blanket rule like if we don´t have gloves, if we don´t have mask we should not work is really* (un) *acceptable and I don´t think that is in keeping with the oath that we all swore as clinicians.”* (Oncologist 9, UK).

However, other oncologists indicated the ethical duty to do no harm to patients. Indicating a practitioner requiring some form of protection (albeit not the ideal full gear) *“is not just about the clinicians, if I expose myself then I expose every other patient that I come in contact with endanger them as well. (By) protecting myself, I am also protecting every patient that I am going to see subsequently after seeing a possible COVID-19 positive patient. So, I think that it is important to not work if you don´t have the tools to keep yourself and everyone that is dependent on you safe.”* (Oncologist 4, SW Nigeria). Furthermore another oncologist stressed the differing circumstance or reality in Nigeria. *“If anyone gets infected, the setting in Nigeria is different. So, the best thing at this point is if you want to continue giving chemo* (therapy) *do so without putting yourself at risk. That* (is) *also covered* (by) *the Hippocratic oath we have sworn to and has been recently amended.”* (Oncologist 3, SW Nigeria).

## Discussion

As mentioned earlier, it is necessary we ensure the solution to a problem is not worse than the problem in itself. Otherwise, more people would be affected by issues other than COVID-19, when the pandemic is over. There is evidence to show that the disruption to health-care services in Guinea, Liberia and Sierra Leone due to the West African Ebola epidemic led to an estimate of up to 10,900 additional malaria deaths [8, 9]. However, there are background challenges to cancer care even before the COVID-19 pandemic. In Nigeria there are only six oncology centres with functioning radiotherapy machines. Therefore, patients may have to travel long distances across states for oncology care. The lockdown imposed by various states further affected the mobility of patients. It is of note that the BBC reported, on the 16^th^of April, that the security forces enforcing the lockdown had killed more people (18) in Nigeria than the Coronavirus did (12) as of the date of the report [10].The Nigerian government and even the private sector needs to improve upon the oncology centre to people ratio, as the IAEA recommends 4-8.1 radiotherapy centres per 1 million people [11, 12].

Patients being unwilling to come for treatment for fear of contracting the disease from health workers is not unwarranted. As of the 20^th^of April, 7 out of the 16 COVID-19 cases in Oyo state work for University College Hospital [13-17]. The health workers are also human. And thus they entertain a reasonable fear of contracting the disease themselves. The incidences raised in the discussion by two oncologists who called the toll free numbers and could not get through is not assuring. It makes the healthcare workers feel they are on their own should they come down with the virus. Factors feeding into the fears are the background resource constraints including inadequate medical facilities and low occupational hazard allowances [18]. Since oncology care demands synergy between fields such as surgery, radiation oncology, medical oncology, radiology and pathology; institutions need to strengthen multi-disciplinary tumour board meetings. However, COVID-19 has further necessitated the need for joint decision making between the disciplines for each patient. This is because every situation needs to be assessed case by case.

The question of appropriate PPE in this COVID-19 pandemic will continue to be an evolving issue which would stretch the boundaries of safety and ethics. This discussion did not particularly reach a consensus. However, most discussants agreed it would be adequate if the practitioners wear a surgical face mask. It would be even better if the patient also wears a mask. Gloves should be reserved for examination and other procedures and not worn for extended periods of time while frequent hand washing, particularly after seeing each patient, is essential. Other challenging considerations and discussions leaders in oncology care would need to address include: What would be the appropriate PPE for radiographers/ radiotherapy technologists? Especially considering they are exposed to multiple patients on a daily basis. What would be the appropriate PPE to wear during brachytherapy? What if the patient is confirmed to be COVID-19 positive? Should such a patient´s treatment cease till he/she is confirmed negative? If one opts to continue treatment, how would the patient be isolated in a resource constrained environment like Nigeria? Can one radiotherapy machine, one chemotherapy suite, and one team of staff be dedicated to treating suspected/confirmed cases?

Patient protection was further stressed by the fact that cancer patients could have their immunity further depressed by chemotherapy, rendering them more susceptible to COVID-19. A study from China by Liang et al shows that COVID-19 positive cancer patients are more likely to have severe events (ventilation or death) compared to non-cancer COVID-19 patients [19]. However, a critical review of the study reveals only 18 cancer patients with COVID-19 were in the sample. Also, the most prevalent cancer type was Lung cancer and the cancer patients were older [19]. As earlier highlighted, the COVID-19 pandemic will raise ethical considerations. Doctors in Italy reported they were faced with the ethical dilemma of deciding who gets a bed and a respirator and who does not. Largely, younger, otherwise healthy patients were prioritized over older patients or those with pre-existing co-morbidities [7, 20].

The COVID-19 incidences and mortalities in Nigeria are relatively low and therefore doctors are not faced with such distressing decisions. However, the depleting resources have raised other ethical circumstances like was alluded to in the discussion. There is currently no consensus on the ethically acceptable stance to take when managing patients in the face of incomplete or absent PPE, as such clinicians must rely on the physician´s oath and the need for self-preservation as they deliver care. The newly revised version of the physician´s oath that oncologist 3 referred to was adopted by the world medical general assembly in 2017. It includes in the pledge “I will attend to my own health, well-being, and abilities in order to provide care of the highest standard [21].” Closely related to ethical issues are legal issues. Could there, possibly, be law suits or compensations due to decisions made by health care workers during this pandemic?

### Solutions

In addition to the recommendation on protective gear above, the discussants proffered other practical solutions that can be achieved in Nigeria.

**Case by case prioritization:** given all the issues expounded above, one can deduce that cancer care cannot proceed unhindered as before the pandemic. One of the oncologists advised care should be prioritized for *“those* (for whom) *it* (would) *actually make a difference in their patient-journey.”* (Oncologist 6, SE Nigeria). Another discussant echoed this by saying *“patients where the yield of treatment is relatively low, the patient who has pancreatic cancer who is for second line treatment of course I would deprioritize that.”* (Oncologist 9, UK).

**Oral/metronomic chemotherapy:** some of the discussants opined patients may benefit from oral drug treatment. However, it should not be done if it would jeopardize treatment outcome.

**Multidisciplinary tumour boards:** most surgeons have stopped elective surgeries. Yet, amongst the oncologists practicing in Nigeria, the ones in centres with strong collaboration with their surgeons through a multidisciplinary team are more confident of getting patients who would benefit from upfront surgery to surgery. It would also be necessary to carry along the intensive care unit with any decision to operate as *“there is no point of having surgery if your ICU has then decided to prioritize your COVID patients and there is no bed.”* (Oncologist 10, UK).

**Screening questionnaire and temperature:** to curb the spread of COVID-19 within the hospital, all patients should be subjected to a screening questionnaire and temperature check. Though this is not full proof; it could help to redirect cases to the center´s COVID response team.

**Spacing:** similarly, patients should not sit closely packed in waiting areas or even during chemotherapy as before the pandemic. The recommended 6 feet social distancing should be upheld. Furthermore, examination procedures should only be done if absolutely necessary.

**Hand gloves vs hand washing vs hand sanitizers:** gloves should not be worn for extended periods. As it creates a false sense of security while possibly spreading germs. *“It is safer to tell* (hospital workers) *to wash their hands than to wear gloves. In my clinic for instance, you see* (health care workers) *who wear gloves and touch everything with those gloves It would actually be safer for them not to wear* (gloves) (but) *wash their hands regularly.”* (Oncologist 4, SW Nigeria) An oncologist shared an innovation used to get people to wash hands in his/her centre; where *“we have someone who* (rings) *a bell every ten minutes to remind everyone to wash their hands.”*Hand sanitizers are also adequate but not better than hand washing with soap and water for at least 20 seconds.

**Appointment systems:** hospitals need to change appointment systems, this may be a gain from COVID-19. Patients should be given appointments of blocks of 15-30 minute periods, rather than appointments based on a date which results in over-crowding the waiting room. Some oncologists also revealed that in the UK, apart from the multiple appointment blocks, patients for procedures are asked to wait at home or in their car. When the centre is absolutely ready to start the procedure, the patients are then called.

**Telemedicine:** patients who are on follow-up and patients that only require a prescription do not have to appear physically at the clinic or hospital. *“One of the good things that* (will) *come out of COVID is that we would begin to have more telemedicine in Nigeria and many centres* (will) *use apps and websites* (to) *keep in touch with their patients, follow up and monitor side effects. I think more of that will come into clinical oncology.”* (Oncologist 4, SW Nigeria).

**Advocacy for patient mobility:** in response to the fact that the lockdowns and curfews are negatively affecting cancer patient´s ability to travel for care, it was advised the expertise of lobbyists and advocates are employed. Stating that *“if our governors in [certain states] are saying absolutely nobody moves then that´s not very good policy. It would be important to get policy makers or administrators to get the message to them that yes, there is COVID but people also have cancer, and people also have medical problems that need to be resolved and they cannot put all those people under this kind of curfew.”* (Oncologist 11, USA).

## Conclusion

The COVID-19 pandemic does pose threats to continuing oncological care with myriads of considerations and challenges. Even within the country the issues, challenges and considerations are not entirely similar. As such there is no straightforward answer to the question “what do we do?” Only determined and practical approaches to “risk reduce” while being practically cautious can ensure cancer care continues albeit to a limited degree.
